# Renal hyperparathyroidism: comparative outcomes of parathyroidectomy in two regional centres over 14 years

**DOI:** 10.1308/rcsann.2025.0112

**Published:** 2026-01-12

**Authors:** S Michael, N Matias, O Alwani, W Matkin, E Solomon, A Lively, D Ricardo, M Ahmed, Z Moinuddin, T Augustine, R Chinnadurai, H Doran

**Affiliations:** ^1^ University of Manchester, UK; ^2^ Manchester University NHS Foundation Trust, UK; ^3^ Northern Care Alliance NHS Foundation Trust, UK

**Keywords:** Parathyroid, Renal hyperparathyroid, Parathyroidectomy

## Abstract

**Introduction:**

Renal hyperparathyroidism is a common complication of chronic kidney disease, often requiring parathyroidectomy (PTX) when medical therapy fails. Following surgical intervention, recurrence and postoperative complications, in particular hypocalcaemia, are variable. This study compares post-PTX outcomes between two UK centres, focusing on recurrence rates, length of stay (LOS) and factors influencing postoperative intravenous (IV) calcium administration.

**Methods:**

A retrospective cohort study was conducted across two centres from 2008–2022. Demographic, biochemical, and clinical factors were analysed, with primary outcomes being disease recurrence and postoperative IV calcium requirement.

**Results:**

In total, 114 patients were included: 66 from centre A and 48 from centre B. Some 65.2% underwent total parathyroidectomy in centre A vs 60.4% in centre B. The remainder were subtotal parathyroidectomies. Total recurrence was higher in centre A (34.8%) than centre B (20.8%) and a longer LOS was seen in centre A (median 5.5 days vs 3 days, *p *= 0.007). IV calcium use was higher in centre B (35.4% vs 24.2%, *p *= 0.194), although not statistically significant. Predictors of recurrence were limited, with preloading with alfacalcidol being protective in a univariate model (hazard ratio [HR] 0.33, *p *= 0.005). Higher postoperative phosphate, parathyroid hormone and alkaline phosphate levels were significant predictors of postoperative IV calcium in multivariate analysis (*p *< 0.05).

**Conclusions:**

Significant differences in recurrence and LOS suggest that preoperative disease burden and perioperative management strategies influence outcomes. The higher recurrence at centre A may be linked to disease severity, while the shorter LOS and higher IV calcium use at centre B may reflect different pre- and postoperative care approaches. These findings highlight the need for careful management and identification of factors which may impact outcomes.

## Introduction

Renal hyperparathyroidism is a serious and common complication of chronic kidney disease (CKD) characterised by the overproduction of parathyroid hormone (PTH) in response to disordered metabolism of calcium, phosphate and vitamin D. Although medical therapies including cinacalcet have improved disease management, many patients remain refractory to pharmacological management and need surgical intervention.^1^ Parathyroidectomy (PTX), either total or subtotal, has been the main approach for this. However, despite the effectiveness of surgical management, recurrence remains a significant concern.

Recurrence, defined by elevated PTH levels following an initial postoperative normalisation or the need for further operative or medical management, has been observed in up to 20% of patients undergoing subtotal parathyroidectomy (SPTX) and in 3.8–9.6% of those undergoing total parathyroidectomy (TPTX).^2–8^ In some cohorts, recurrence rates have reached as high as 22% in dialysis-dependent patients.^9^ Some studies also highlight better disease control following TPTX.^10–12^

Multiple factors influence the recurrence of renal hyperparathyroidism following parathyroidectomy. Studies have identified high preoperative PTH levels, dialysis vintage, and elevated postoperative PTH and phosphate levels as key predictors.^8,9,13^ In addition, incomplete resection of parathyroid tissue and the presence of ectopic or supernumerary glands can contribute to this.^5^ Time to recurrence also varies, with some patients experiencing biochemical recurrence within months, while others develop clinically significant disease years after surgery.

Although operative management can offer good long-term control, perioperative care can be challenging, with rapid and profound hypocalcaemia due to accelerated bone remineralisation following PTX. The incidence of hypocalcaemia varies widely, ranging from 27.4% to 87.8% in patients undergoing surgery for renal hyperparathyroidism, with younger age, high preoperative alkaline phosphatase (ALP) and low preoperative serum calcium consistently identified as significant predictors.^6,14–17^ Additional risk factors include increased weight of resected glands and prolonged hyperparathyroidism before surgery.^18^ Despite its frequency, the severity of hypocalcaemia and its duration are variable, often requiring intensive calcium and vitamin D supplementation during the postoperative period to mitigate clinical symptoms and avoid complications such as cardiac arrhythmias, and to reduce the length of stay (LOS). Understanding the risk factors and predictors underlying hypocalcaemia is critical to improving preoperative preparation and postoperative management. One study of 84 patients found that vitamin D preloading was not able to prevent hypocalcaemia and did not impact the length of treatment.^19^

The aim of this study was to compare the post-PTX outcomes of two regional tertiary centres in the UK comparing LOS, incidence of IV calcium and recurrences. In addition to optimising case ascertainment, the two regional centres were chosen because of differences in the disease patterns and management protocols at both centres, with one centre being a primary endocrine centre and the other being a primary transplant centre, and to compare and contrast differences while keeping a similar patient demographic, given their close geographical location. The secondary outcomes of this paper were to explore factors predicting recurrence or postoperative IV calcium.

## Methods

### Patient selection

This was a retrospective cohort study conducted across two regional tertiary centres, with data collected over a 14-year period from January 2008 to December 2022 simultaneously. All patients with CKD stage 4/5 or end-stage kidney disease receiving renal replacement therapy undergoing PTX for hyperparathyroidism during this time frame were included, as well as all patients who had a renal transplant whether functioning or not. These patients were identified through the electronic patient record in both hospitals; criteria for intervention in both cohorts were the same, with surgery being offered for symptomatic hypercalcaemia, renal disease such as renal stones or nephrocalcinosis, osteoporosis or fragility fractures, and failure or intolerance of medical management. Patients with primary hyperparathyroidism, those undergoing redo operations, or those with incomplete medical records were excluded from the analysis. Patients with other histology such as having a thyroidectomy were also excluded. Demographic information, such as age, sex and ethnicity, was gathered for each patient. Preloading was also recorded between the sites; preloading was standard in centre B with a protocol advising all patients to be preloaded with 5μg alfacalcidol for 5 days. Centre A was preloaded at the surgeon’s discretion with no protocol for this. Surgical details including the type of operation, SPTX or TPTX, were recorded, along with histological findings confirming hyperplastic parathyroid tissue. Biochemical data, including preoperative and postoperative levels of PTH, calcium, phosphate and ALP, were obtained to evaluate biochemical resolution and identify predictors of recurrence.

### Definitions

The study included clinical details on dialysis duration and type, primary renal disease, history of kidney transplantation and outcomes; recurrence of hyperparathyroidism, the incidence of postoperative IV calcium, LOS, postoperative cardiovascular events and mortality. The surgical procedure was made in consultation with renal colleagues and guided by renal status and intraoperative findings (burden of disease). Patients with a functioning renal transplant, or those on dialysis with the prospect of future transplantation, were generally offered SPTX, whereas those unlikely to be transplant candidates, owing to factors such as severe vascular calcification, advanced cardiac disease, poor compliance, limited hospital engagement or prior malignancy, were more often considered for TPTX. Intraoperative assessment also influenced the decision, with TPTX more likely if all four glands were grossly diseased. The final decision on this was at the discretion of the operating surgeon. Clinical recurrence was defined as any recurrence needing further medical management or operative management after a minimum period of 12 months after surgery. Biochemical recurrence was defined as persistent rise in serum PTH above the normal after an initial normal PTH (defined as a PTH >6.9pmol/ml) after a minimum period of 12 months. Postoperative IV calcium administration was defined as any patient requiring IV calcium replacement during the postoperative period prior to discharge.

### Ethical considerations

This study was conducted as two retrospective audits at each respective hospital and was registered as a clinical audit in both sites.

### Statistical analysis

Data analysis was performed using SPSS version 28. Statistical comparisons of categorical variables were made using chi-squared tests, and continuous variables were analysed with the Mann–Whitney *U* test for non-parametric variables and Student’s *t*-test for parametric variables. Univariate and multivariate Cox regression analysis was used to identify risk factors associated with recurrence and binary logistic regression analysis was used to assess predictors of postoperative IV calcium administration. A *p*-value <0.05 was considered statistically significant.

## Results

A total of 114 patients were included in the study: 66 patients treated at centre A and 48 patients treated at centre B (Supplementary Figure 1, available online). Table 1 provides a comparative summary of the demographic and clinical characteristics for the two cohorts. There was a similar gender split and mean age in both centres. Mean follow-up was 7 years for centre A and 6.6 years for centre B.

**Table 1:** Comparison of demographic data across the two centres

Variable	Total (*n *= 114)	Centre A (*n *= 66)	Centre B (*n *= 48)	*p*-value
Age, years (mean) [sd]	52.8 [13.57]	51.8 [13.89]	54.10 [13.13]	0.374
Sex, male	63 (55.3)	33 (50)	30 (62.5)	0.185
Ethnicity				**0.003**
Asian	10 (8.8)	5 (7.6)	5 (10.4)	
Black	14 (12.3)	13 (19.7)	1 (2.1)	
White	84 (73.7)	42 (63.6)	42 (87.5)	
Other	6 (5.3)	6 (9.1)	0	
Operation				0.605
Subtotal	42 (36.8)	23 (34.8)	19 (39.6)	
Total	72 (63.2)	43 (65.2)	29 (60.4)	
Medication				
Cinacalcet preoperatively	49 (43.0)	27 (40.9)	22 (45.8)	0.600
Alfacalcidol preoperatively	64 (56.1)	39 (59.1)	25 (52.1)	0.457
Preloaded?	48 (42.1)	10 (15.2)	38 (79.2)	**<0**.**001**
Renal status at time of surgery				0.250
Predialysis	15 (13.2)	11 (16.7)	4 (8.3)	
Working transplant	35 (30.7)	17 (25.8)	18 (37.5)	
Dialysis	64 (56.1)	38 (57.6)	26 (54.2)	
Biochemical markers				
Preoperative calcium, mmol/L (median) [IQR]	2.65 [0.41]	2.59 [0.34]	2.79 [0.31]	**0**.**002**
Preoperative parathyroid hormone, pmol/ml (median) [IQR]	71.4 [117.60]	104.30 [142.00]	49.90 [113.70]	**0**.**010**

Categorical variables are expressed as number (%) and *p*-value by chi-squared test. Continuous variables are expressed as mean (sd) and *p*-value by Student’s *t*-test for parametric variables (age) and median (interquartile range) with Mann–Whitney *U* tests for non-parametric (preoperative calcium and parathyroid hormone). Significant *p*-values are shown in bold.

IQR = interquartile range.

Significant differences in ethnicity distribution were observed (*p *= 0.003), with centre B having a higher proportion of White patients (87.5% vs 63.6%) and fewer Black patients (2.1% vs 19.7%) compared with centre A. There was also a notable disparity in alfacalcidol preloading, with centre B reporting a significantly higher rate of use because of a standardised protocol (79.2% vs 15.2%, *p *< 0.001).

In terms of clinical details, a higher proportion of patients at centre A were on dialysis (57.6% vs 54.2%), whereas the proportion of patients with working kidney transplants was higher in centre B (25.8% vs 37.5%). Preoperative biochemical parameters revealed a significantly higher median calcium level (2.79 vs 2.59mmol/L, *p *= 0.002) but a lower median PTH level (49.90 vs 104.30pmol/ml, *p *= 0.010) in patients at centre B compared with centre A. In centre A, 65.2% of patients underwent TPTX compared with 60.4% in centre B. The remainder were SPTX. Two patients (1.8%) had a parathyroid adenoma, and the remaining 112 (98.2%) had parathyroid hyperplasia on histology.

Postoperative outcomes are summarised in Table 2. Centre B demonstrated a significantly shorter average hospital LOS (3 vs 5.5 days, *p *= 0.007) compared with centre A. Clinical recurrence requiring surgical reintervention or cinacalcet therapy was observed in 10.6% of centre A patients, whereas there were no recurrences reported in centre B (*p *= 0.020). Biochemical recurrence was higher in centre A (34.8%) than in centre B (20.8%), although this difference did not reach statistical significance (*p *= 0.103). Total recurrence was higher in centre A (39.4%) compared with centre B (20.8%) and this result was significant (*p *= 0.035). Among the study cohort, 26 patients failed to achieve normalisation of PTH immediately following surgery. Of these, 13 patients (50%) had undergone subtotal parathyroidectomy. Clinical recurrence was observed in two patients: one required reoperation and the other was managed with cinacalcet, 12 months from the date of their operation. Biochemical recurrence occurred in 24 patients; in two of the 26 cases, PTH levels subsequently normalised after 12 months.

**Table 2:** Comparison of postoperative outcome data between the two centres

Outcome	Centre A (*n *= 66)	Centre B (*n *= 48)	*p*-value
IV calcium postoperatively	16 (24.2)	17 (35.4)	0.194
Day 1 calcium, mmol/L (median) [IQR]	2.34 [0.47]	2.49 [0.39]	**<0**.**001**
Day 1 parathyroid hormone, pmol/ml (median) [IQR]	2.50 [9.00]	1.20 [5.00]	**0**.**005**
Clinical recurrence (operative or cinacalcet)	7 (10.6)	0	**0**.**020**
Biochemical recurrence	23 (34.8)	10 (20.8)	0.103
Total recurrence	26 (39.4)	10 (20.8)	**0**.**035**
Mortality (other causes)	23 (34.8)	15 (31.3)	0.687
Length of stay,^a^ (days) (median) [IQR]	5.50 [16.00]	3.00 [4.00]	**0**.**007**

Categorical variables are expressed as number (%) and *p*-value by chi-squared test. Continuous variables are expressed as median (interquartile range) with Mann–Whitney *U* tests for non-parametric (day 1 calcium, day 1 parathyroid hormone and length of stay). Significant *p*-values are shown in bold.

^a^Four patients were excluded from length of stay analysis owing to other factors influencing their hospital stay such as other concurrent surgical procedures or other medical comorbidities.

IQR = interquartile range; IV = intravenous

The incidence of postoperative IV calcium was higher at centre B (35.4% vs 24.2%), but again, this difference was not statistically significant (*p *= 0.194).

**Figure 1:**
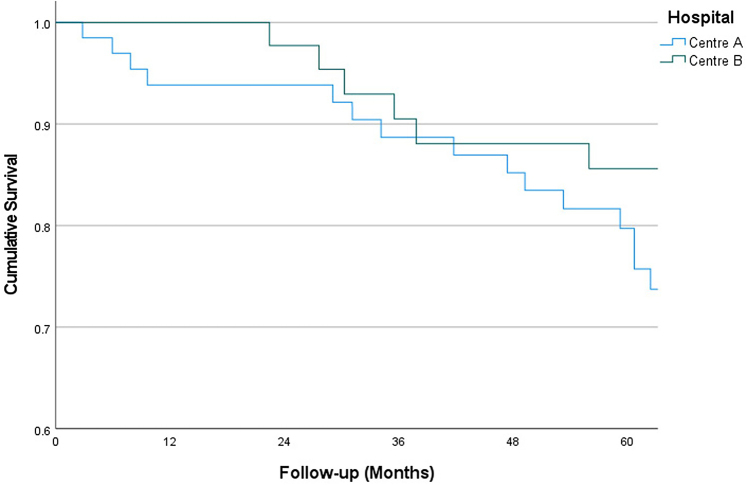
Kaplan–Meier graph showing 5-year mortality across both centres

Mortality rates, none of which were immediately postoperative, were comparable between the two centres (34.8% at centre A vs 31.3% at centre B, *p *= 0.687). The 2-year mortality across the cohort was 4.4% (five patients), and the 5-year mortality was 15.8%. This is presented across both centres in Figure 1.

Table 3 shows factors predicting recurrence after PTX. The results showed that factors such as age, preoperative PTH, preoperative calcium, preoperative phosphate, postoperative calcium and postoperative phosphate were not significant predictors of recurrence. Type of operation, and clinical factors such as being on dialysis or having a functioning transplant, did not predict recurrence. Only preloading with alfacalcidol was shown to be significant in the univariate analysis.

**Table 3:** Univariate Cox regression on factors predicting recurrence

Variable	Univariate HR (CI)	*p*-value
Age	1.02 (0.99–1.04)	0.278
Preoperative parathyroid hormone, pmol/ml	1.00 (0.99–1.00)	0.588
Preoperative calcium, mmol/L	1.24 (0.42–3.68)	0.704
Preoperative phosphate, mmol/L	0.79 (0.48–1.31)	0.359
Preoperative ALP, mmol/L	1.00 (0.99–1.00)	0.793
Postoperative calcium, mmol/L	0.84 (0.29–2.42)	0.751
Postoperative phosphate, mmol/L	1.08 (0.62–1.87)	0.786
Postoperative parathyroid hormone, pmol/ml	1.01 (0.99–1.02)	0.292
Postoperative ALP, mmol/L	1.00 (0.99–1.00)	0.495
Hospital	0.52 (0.25–1.10)	0.085
Sex (female vs male)	0.90 (0.47–1.74)	0.758
Subtotal vs total	1.19 (0.60–2.35)	0.626
Cinacalcet preoperatively?	1.33 (0.67–2.56)	0.392
Alfacalcidol preoperatively?	0.81 (0.41–1.59)	0.541
Preloaded?	0.33 (0.16–0.72)	**0**.**005**
Transplant?	0.72 (0.37–1.43)	0.350
Dialysis?	1.06 (0.54–2.10)	0.864
IV calcium given?	0.71 (0.32–1.56)	0.392

ALP = alkaline phosphatase; CI = confidence interval; HR = hazard ratio; IV, intravenous

These results were later focused to analyse only those patients who had been receiving dialysis (Supplementary Table 1, available online). The results were largely consistent with the main cohort; preoperative PTH, preoperative calcium and postoperative calcium did not show a significant predictive value for recurrence. Increased age and being preloaded did predict for lower recurrence in this subgroup in the univariate analysis, but this finding did not carry through to the multivariate analysis.

Factors predicting IV calcium requirement after PTX are shown in Table 4. In univariate analysis, several factors were significantly associated with the outcome, including preoperative PTH, phosphate, ALP; postoperative calcium, phosphate, PTH, ALP; subtotal vs total surgery and dialysis. However, in multivariate analysis, only postoperative phosphate (odds ratio [OR] 11.287, *p *= 0.007), postoperative PTH (OR 0.785, *p *= 0.008) and postoperative ALP (OR 1.008, *p *= 0.025) remained significant predictors. Significant *p*-values are shown in bold.

**Table 4:** Univariate and multivariate logistic regression for factors that predict intravenous calcium administration

Variable	Univariate OR (CI)	*p*-value	Multivariate OR (CI)	*p*-value
Age	0.98 (0.95–1.01)	0.223		
Preoperative parathyroid hormone, pmol/ml	1.01 (1.00–1.01)	**0**.**007**	1.00 (0.99-1.01)	0.876
Preoperative calcium, mmol/L	0.27 (0.06–1.18)	0.081		
Preoperative phosphate, mmol/L	1.93 (1.03–3.59)	**0**.**039**	0.41 (0.10–1.63)	0.203
Preoperative ALP, mmol/L	1.02 (1.00–1.04)	**0**.**004**	0.99 (0.99–1.00)	0.143
Postoperative calcium, mmol/L)	0.10 (0.03–0.41)	**0**.**001**	0.17 (0.02–1.31)	0.088
Postoperative phosphate, mmol/L	2.88 (1.34–6.22)	**0**.**007**	11.29 (1.92–66.27)	**0**.**007**
Postoperative parathyroid hormone, pmol/ml	0.89 (0.79–1.00)	**0**.**044**	0.79 (0.66–0.94)	**0**.**008**
Postoperative ALP, mmol/L	1.03 (1.00–1.01)	**0**.**003**	1.01 (1.00–1.01)	**0**.**025**
Hospital	1.71 (0.76–3.88)	0.196		
Sex (female vs male)	0.81 (0.36–1.82)	0.608		
Subtotal vs total	3.60 (1.34–9.66)	**0**.**011**	3.28 (0.88–12.23)	0.077
Cinacalcet preoperatively?	0.68 (0.30–1.56)	0.363		
Alfacalcidol preoperatively?	2.24 (0.95–5.31)	0.066		
Preloaded?	1.44 (0.64–3.26)	0.380		
Transplant?	0.63 (0.28–1.43)	0.273		
Dialysis?	3.98 (1.48–10.66)	**0**.**006**	2.75 (0.55–13.80)	0.219

Significant *p*-values are shown in bold.ALP = alkaline phosphatase; CI = confidence interval; OR = odds ratio.

A further subgroup analysis is shown in Supplementary Table 2 (available online) for dialysis-only patients and factors that predict postoperative IV calcium administration. In univariate analysis, postoperative PTH (OR 0.87, *p *= 0.041), hospital (OR 3.02, *p *= 0.040) and subtotal vs total surgery (OR 3.76, *p *= 0.034) were significantly associated with the outcome. However, in multivariate analysis, none of these factors remained statistically significant, although postoperative PTH (OR 0.89, *p *= 0.095) and subtotal vs total surgery (OR 2.78, *p *= 0.129) showed a trend towards significance. Other factors, including age, sex, preoperative and postoperative biochemical markers, dialysis history and medication use, were not significantly associated with the outcome in either analysis.

## Discussion

This study aimed to compare the outcomes of PTX for renal hyperparathyroidism across two regional tertiary centres, focusing on factors influencing recurrence and the incidence of postoperative IV calcium. A significant strength of this study is that, to our knowledge, it represents one of the largest UK data sets of patients with renal hyperparathyroidism with extensive biochemical and clinical data. It is a varied cohort of patients, including those with predialysis CKD, those receiving dialysis and those with transplants. This is in contrast to other published studies, which mainly feature only patients receiving dialysis.^6,12,20–22^ Our cohort also features a higher proportion of patients with working renal transplants compared with other similar studies (34% vs 17.4%).^23^

A key finding from this study was the significant difference in postoperative outcomes between the two centres, particularly with respect to recurrence rates and LOS. Centre A exhibited a notably higher recurrence rate requiring surgical reintervention or medical therapy (10.6%) compared with centre B, which reported no cases of recurrence. Although biochemical recurrence was more frequent at centre A (34.8%) than at centre B (20.8%), this difference did not reach statistical significance. The British Association of Endocrine and Thyroid Surgeons (BAETS) national audit reports biochemical recurrence in this cohort of between 2.6% and 23.1% depending on surgical approach used.^24^

The higher overall recurrence rates at centre A may be multifactorial, potentially influenced by the higher preoperative PTH levels observed in its patient cohort, suggesting a more severe disease burden. Patients in centre A also had a higher burden of dialysis patients as well as patients with a higher preoperative PTH, potentially suggesting worse disease. This may suggest a need for earlier referral to surgical teams in centre A, which may further impact and reduce LOS and recurrence rates. In addition, differences in patient demographics, such as ethnicity and the proportion of transplant patients, may have contributed to this disparity and warrant further investigation. Notably, the recurrence rates observed in this study align with those reported in the broader literature.^2–9,23^ One important factor that may have influenced recurrence, and which needs further investigation in this population, is surgical practice. It was routine practice in centre B to perform a thymectomy for all parathyroidectomies, but this was not the routine practice for all surgeries in centre A; in the literature this has been shown to almost halve the recurrence rates for patients with renal hyperparathyroidism.^25^

Another finding was the difference in the average LOS between the two centres. Centre B exhibited a shorter LOS (3 days) than centre A (5.5 days). This discrepancy may be attributed to potential variations in postoperative care, but is more likely due to other confounding factors. The higher use of preloaded alfacalcidol at centre B likely contributed to this shorter LOS, although further investigation is needed to confirm this hypothesis.

Preoperative factors, including elevated PTH, calcium, phosphate and time on dialysis, are well-documented predictors of postoperative outcomes following PTX.^8,9,13^ This study has additionally identified that a raised postoperative ALP is a predictor of the need for IV calcium administration, and a higher postoperative phosphate also emerged as a very strong predictor for needing IV calcium postoperatively. These results underscore the importance of assessing these biochemical markers prior to surgery to identify patients at higher risk for postoperative complications. This study also showed a protective effect against recurrences with preloading prior to the surgery; however, this effect was only seen on the univariate analysis and warrants further study with a larger cohort. In contrast to other studies, no significant associations were found between preoperative calcium or phosphate levels and recurrence. There was also no association found between time to dialysis and recurrence in this cohort.

Although the incidence of postoperative IV calcium was higher at centre B (35.4%) than at centre A (24.2%), this difference did not reach statistical significance. This is comparable with the data from the BAETS national report, which notes a 48% rate of early hypocalcaemia postoperatively in this cohort between 2006 and 2020.^24^ However, the trend suggests that patients at centre B may be at greater risk for postoperative IV calcium, which aligns with the higher postoperative levels of ALP observed in this cohort, consistent with previous studies linking elevated ALP with an increased risk of postoperative IV calcium. Another factor that may have impacted the incidence of postoperative IV calcium in both centres is the difference in hospital policy for managing postoperative calcium in this group of patients. In centre A, IV calcium is recommended for all patients with a serum calcium below 1.6mmol/L and for symptomatic patients only below 1.8mmol/L. By contrast, centre B recommends IV calcium replacement in all patients with serum calcium below 2mmol/L, irrespective of symptoms. This is in keeping with the median postoperative calcium for both sites, with centre A having a lower calcium (2.34mmol/L) compared with centre B (2.49mmol/L), suggesting that centre B are treating with IV calcium at higher levels than centre A. Patients above the threshold presented above were given oral calcium instead according to the protocol. In both centres, IV calcium was administered by peripheral cannulas as needed.

Higher preoperative ALP levels are known to be predictive of postoperative IV calcium, because accelerated bone remineralisation can lead to significant drops in calcium levels postoperatively. Interestingly, higher postoperative ALP and phosphate levels were associated with postoperative IV calcium, suggesting that careful monitoring and management of ALP and phosphate during the postoperative period may be beneficial in reducing the risk of these complications by identifying those patients with a high postoperative ALP and phosphate early, and monitoring for signs of profound hypocalcaemia. A lower postoperative PTH also predicted needing IV calcium, which is in keeping the other studies. Crucially, preloading with alfacalcidol was not found to prevent or predict postoperative IV calcium, but may have impacted LOS, again in keeping with other studies.^19^ This is an area that requires further investigation.

### Clinical implications

The results of this study suggest that individualised preoperative assessment and postoperative management are critical in optimising outcomes for patients undergoing PTX for renal hyperparathyroidism. Further study and attention should be given to postoperative ALP and phosphate levels; they serve as a novel predictor for IV calcium usage in this study, but a causal or predictive link cannot be assumed from our findings. Preloading may also have a protective benefit, as seen in the univariate analysis, but is not an independent predictor. The clinical significance of this finding is unclear and warrants further study with a larger sample size. This study also highlights the importance of considering institutional practices and protocols when interpreting PTX outcomes, because variations between centres can significantly impact patient recovery and long-term results.

### Study limitations

Although this study provided valuable insights, there are several limitations that should be acknowledged. First, this was a retrospective cohort study, and as such, there is a potential for selection bias in the inclusion of patients. Second, the study was conducted across two centres with varying practices and patient demographics, which may limit the generalisability of the findings. The centres also differed in exclusion criteria, which may introduce bias into the study. Other complications such as vocal changes were unfortunately not captured in this data set in centre A. In centre B, there were no postoperative haematoma and one partial vocal cord palsy (identified and repaired at time of surgery with some function retained). Furthermore, the relatively small sample size limits the power of some of the comparisons. The statistical findings do not allow for preoperative classification and will need further study to confirm their validity in a larger sample size.

## Conclusions

In conclusion, this study demonstrates significant differences in postoperative outcomes following parathyroidectomy for renal hyperparathyroidism between two regional centres. Centre B showed a lower incidence of recurrence, and a significantly shorter LOS compared with centre A. Overall, postoperative ALP and phosphate emerged as potential predictors of IV calcium administration. These findings highlight the importance of careful preoperative assessment and postoperative management, particularly with regard to biochemical markers such as ALP, in improving long-term outcomes for patients undergoing PTX. This can allow for further identification of patients who need closer monitoring and encourages discussion between renal and endocrine teams looking after these patients. Future studies, particularly prospective, multicentre trials with larger sample sizes, are needed to validate these findings and further identify strategies to predict and minimise recurrence and complications like postoperative IV calcium.

## Competing interests

The author/s declare no competing interests.

## Funding

The authors received no financial support for the research, authorship, and/or publication of this article.

## Ethics approval and consent to participate

Not applicable.

## Conflicts of interest

The authors declare no conflicts of interest.

## Artificial intelligence

The author/s declare that no AI was used to conduct the study or prepare the manuscript.
